# Chemically synthesized CdSe quantum dots induce apoptosis in AGS gastric cancer cells *via* ROS generation

**DOI:** 10.1039/d4na00795f

**Published:** 2024-11-22

**Authors:** L. T. T. Huong, N. P. Hung, N. T. Ha, N. T. Luyen, N. T. Hien, N. X. Ca, N. T. M. Thuy

**Affiliations:** a Faculty of Biotechnology, TNU-Thai Nguyen University of Sciences Vietnam; b Center for Interdisciplinary Science and Education, Thai Nguyen University Vietnam; c Institute of Science and Technology, TNU-Thai Nguyen University of Sciences Vietnam; d Faculty of Physics, TNU-University of Education Thai Nguyen Vietnam thuyntm@tnue.edu.vn; e Medical University of Lublin Poland

## Abstract

CdSe quantum dots (QDs) with size in the range of 3.5–5.8 nm and a zinc blende (ZB) crystal structure were synthesized by the wet chemical method. The morphology of the synthesized QDs was assessed by transmission electron microscopy (TEM). The structural and optical properties were characterized by X-ray diffraction (XRD), absorption spectroscopy (Abs) and photoluminescence (PL) spectroscopy. The anti-cancer activity of CdSe QDs was investigated on AGS gastric cancer cells through cell viability screening (MTT assay), cell cycle and apoptosis analysis using flow cytometry. The generation of reactive oxygen species (ROS) was analyzed using the cell fluorescence staining method with H2DCFDA. Three QD series of CdSe1 (3.5 nm), CdSe2 (4.7 nm) and CdSe3 (5.8 nm) have been selected to study their effects on the extermination of stomach cancer cells. The CdSe QDs all exhibited the potential to induce toxicity to cells at concentrations ranging from 5 to 20 μg mL^−1^. CdSe2 demonstrated a significant impact on cell proliferation compared to the CdSe1 and CdSe3 forms (*p* < 0.01). CdSe QDs caused cell cycle arrest, leading to the accumulation of cells in the G0/G1 phase, while also increasing the rate of apoptosis compared to the control (*p* < 0.01). More importantly, it has been demonstrated that CdSe QDs promote excessive production of ROS in AGS cells, which is believed to be the cause of apoptosis and the reduction of cell proliferation. These data suggest that CdSe QDs are a good candidate for combating gastric cancer cells.

## Abstract 2

Effect of CdSe QDs on proliferation and morphology of AGS gastric cancer cells.

## Graphical abstract

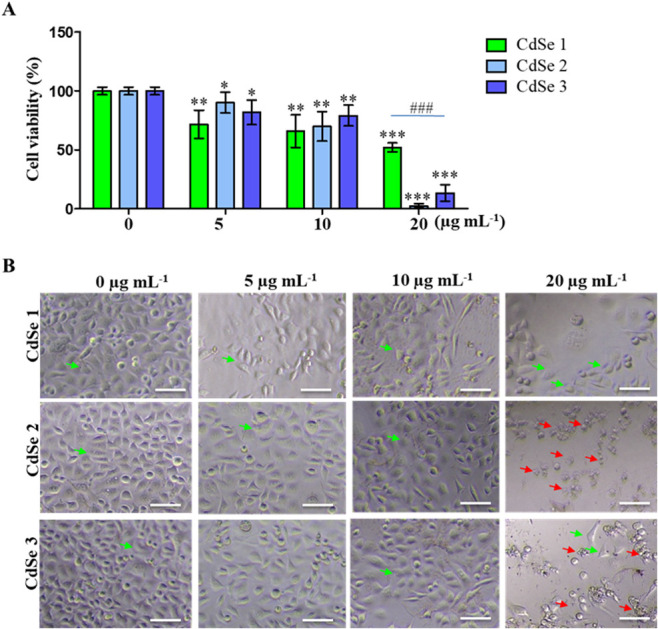

## 1 Introduction

Stomach cancer is the fourth most common cancer worldwide, and the early detection and effective treatment of stomach cancer cells are always challenging. Stomach cancer is a malignant tumor that develops in the lining of the stomach. It often begins in the innermost layer, the mucosa, and can spread to deeper layers as it grows. Risk factors for stomach cancer include a diet high in smoked and pickled foods, smoking, *Helicobacter pylori* infection, and a family history of the disease.^1–3^ According to the latest statistics from Globocan, in 2020 alone, there were over one million new cases and approximately 800 000 deaths due to stomach cancer.^1^ Accurate targeting and selective toxicity are challenges encountered in the development of current cancer therapies. Treatment depends on the stage of the cancer and may include surgery, chemotherapy, radiation therapy, or targeted drug therapy. Preventive measures include maintaining a healthy diet, avoiding tobacco, and regular medical checkups for those at higher risk. Medical research advances improve survival rates, especially when the cancer is caught early. Despite these improvements, stomach cancer remains a serious health issue worldwide.

In recent years, the application of semiconductor quantum dots (SQDs) in medicine has attracted strong attention. The significant applications of SQDs in the biomedical field include fluorescent labeling of cells, tissues, and organs.^2,3^ One of the primary uses of SQDs in medicine is in imaging and diagnostics. Due to their unique ability to emit different colors when exposed to light, SQDs are used as fluorescent probes in bioimaging, allowing for the detailed visualization of biological tissues and cells. This is especially helpful in cancer detection, as SQDs can be engineered to bind to specific cancer cells, making them easier to identify. Additionally, SQDs are being explored for their potential in drug delivery systems, where they can be tailored to carry therapeutic agents directly to targeted cells, enhancing treatment precision and reducing side effects. They are also being used in developing biosensors, capable of detecting biomarkers for various diseases, including cardiovascular and infectious diseases. Furthermore, SQDs' capacity for multiplexing—simultaneously tracking multiple biological processes—opens up new possibilities for advanced diagnostics and personalized medicine. QDs are widely applied in medicine for drug delivery, antibacterial applications,^4^ cancer detection,^5^ in tumor-bearing mice (xenograft), and for detecting the presence of Bisphenol-A, a critical factor contributing to endocrine disorders in the body.^6^ Additionally, CdSe QDs are effectively utilized in detecting toxins, antibiotic residues, and contaminating microorganisms in food.^7^

Different types of QDs, such as carbon QDs, graphene QDs, CdSe QDs, or CdTe QDs, exhibit unique physicochemical properties, leading to diverse and rich biological activities, such as antibacterial activity^8^ and antifungal activity,^9,10^ which are effectively applied in food preservation.^11,12^ With their high luminescence and sensitivity, QDs are also used as biosensors to locate or monitor changes in the position of target molecules within cells, with applications in cancer diagnostics.^13,14^ Based on their fluorescence imaging capabilities, QDs are applied in research for detecting changes in the morphology, size of tumor masses, as well as assessing the invasion of cancer cells.^15^ QDs are used as biomarkers to observe the interactions between different cells in gastric cancer.^16^ With their photothermal properties, QDs have been reported to possess the capability to target gastric cancer cells and reduce side effects in *in vivo* models of gastric cancer.^17^ QDs have been reported to be able to combine with monoclonal antibodies such as HER2, PDL-1 or EGFR for early cancer detection.^17,18^ Concurrently, combining QDs with cancer treatment drugs such as 5-FU, cisplatin or docetaxel or doxorubicin and promising biomarkers creates complexes for the precise targeted therapy of cancer cells.^19,20^ Recently, the toxicity of CdSe QDs to liver cancer cells^21^ and breast cancer cells^22^ has been studied. However, data on the impact of QDs on gastric cancer cells are still limited. Furthermore, there are also concerns about the toxicity of SQDs, so studies are still underway to ensure their safe application under clinical conditions. This research aims to determine the optical characteristics and cytotoxicity against stomach cancer cells of chemically synthesized CdSe QDs.

## 2 Experimental

### 2.1 Materials

Cadmium oxide (CdO, 99.99%, powder), 1-octadecene (ODE, 90%), oleic acid (OA, 90%), selenium (Se, 99.99%, powder), toluene (99.8%), isopropanol (99.7%), tri-*n*-octylphosphine (TOP, 97%), MTT reagent, dimethyl sulfoxide (DMSO), and 2′,7′-dichlorodihydrofluorescein diacetate (H2DCFDA) were purchased from Sigma-Aldrich, France. All chemicals were used without further purification. Cell culture medium RPMI and penicillin–streptomycin antibiotic solution were supplied by Thermo Fisher. AGS gastric cancer cell line obtained from the laboratory of Inserm U1312 BRIC – Bordeaux, France was used for biological activity assay of the synthesized CdSe QDs.

### 2.2 Synthesis of CdSe QDs

One-pot synthesis was used to fabricate CdSe QDs by modifying the method reported in ref. 23. Briefly, a mixture of CdO (128 mg), OA (5 mL), and ODE (30 mL) in a three-neck flask was heated to 220 °C and stirred under a nitrogen flow. At this point, the stock solution of Se was separately and rapidly injected. Se precursor solution was previously prepared by dissolving Se in TOP and ODE at 100 °C under nitrogen with constant stirring, respectively. After the injection of the Se solution, the reaction mixture was kept at 220 °C for 2–180 min. The reacted solution containing CdSe QDs was then cooled down to room temperature. After centrifuging the solution in isopropanol at a speed of 15 000 rpm for 10 min and removing the supernatant, the collected sediment of CdSe QDs was redispersed in toluene.

### 2.3 Characterization

The crystal structure of NCs was checked by using X-ray diffraction (XRD, Siemens D5005 diffractometer) equipped with a Cu K_α_ radiation source. The particle shape and size were determined by transmission electron microscopy (TEM, Joel-JEM 1010) operating at 80 kV. Ultraviolet-visible (UV-vis) absorption spectra were recorded by using a V-770 (Varian-Cary) spectrophotometer. Steady-state photoluminescence (SSPL) measurements were performed on a spectrometer (Horiba, iHR550) by using a 355 nm pulsed laser as an excitation source.

### 2.4 Cell culture and treatment with CdSe quantum dots

A quantity of 0.01 × 10^6^ AGS cells were cultured in 0.1 mL RPMI 1640 medium per well in a 96-well plate for 24 hours to allow cell adhesion to the plate surface. Subsequently, new culture media containing CdSe QDs at concentrations ranging from 5 to 20 μg mL^−1^ were added to replace the old medium for 24 hours. Changes in cell morphology due to the impact of quantum dots were observed using a Nikon Ts2 inverted microscope (NIKON, Japan). Next, the culture medium was replaced with a new medium containing MTT reagent (3-(4,5-dimethylthiazol-2-yl)-2,5-diphenyl-2*H*-tetrazolium bromide) at a concentration of 5 mg mL^−1^ for 4 hours. Subsequently, 100 μL of DMSO was added to dissolve the purple crystals formed from the MTT transformation. Cell proliferation of the treated cells compared to the control cells was determined by measuring the OD values using the Multiskan Sky Microplate Spectrophotometer (Thermo Fisher) and applying the formula:% cell viability = (OD treatment/OD control) × 100%

### 2.5 Analysis of the influence of CdSe QDs on cell cycle and apoptosis by flow cytometry

A quantity of 0.5 × 10^6^ cells was cultured in 1 mL of RPMI 1640 medium per well in a 6-well plate for 24 hours. Subsequently, a new medium containing CdSe QDs at concentrations ranging from 5 to 20 μg mL^−1^ was added, replacing the old medium, and incubated at 37 °C with 5% CO_2_. After 24 hours of treatment with CdSe QDs, the cells were collected by incubating with 0.5 mL of 0.25% trypsin for 3 minutes and centrifuged at 1300 rpm for 5 minutes. The collected cells were fixed in 70% ethanol at −20 °C overnight. Subsequently, the cells were stained with a 20 μg mL^−1^ concentration of PI solution in PBS for 1 hour at room temperature. Next, the cell cycle and apoptosis were analyzed by flow cytometry (BD AccuriTM C6 Plus, BD BioSciences, USA).

### 2.6 Analysis of reactive oxygen species generation

AGS cells (0.05 × 10^6^ cells) were cultured in 0.5 mL of medium for 24 hours at 37 °C with 5% CO_2_. Subsequently, the cells were cultured in a new medium containing CdSe QDs at concentrations ranging from 5 to 20 μg mL^−1^ for 24 hours. The culture medium was then completely removed, and the dish surface was washed three times with PBS 1× buffer. Next, a new medium containing 10 μg mL^−1^ H2DCFDA was added for 30 minutes at room temperature. Cells were then imaged using a NIKON Ts2 fluorescence microscope (NIKON, Japan) with an FITC (green) filter, and cell shapes were captured under bright-field illumination. The percentage of cells generating reactive oxygen species (ROS) was determined by dividing the number of green cells by the total number of cells in the contrast phase.

## 3 Results and discussion

**Fig. 1:**
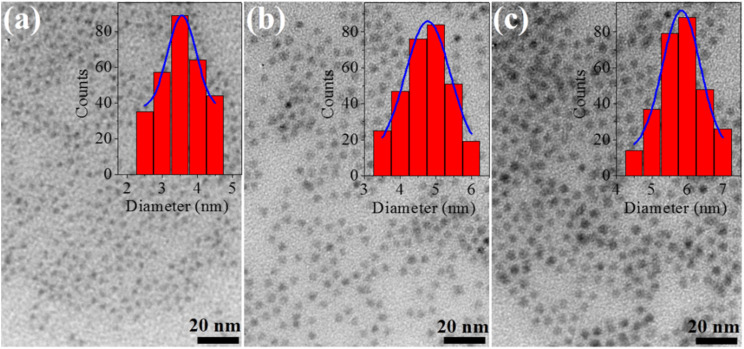
TEM of CdSe QDs at reaction time: (a) 2 min, (b) 30 min, (c) 180 min.

The CdSe QDs were fabricated at 2, 30 and 180 minutes corresponding to the notations CdSe1, CdSe2 and CdSe3. They were selected to study their effects on killing gastric cancer cells. TEM images of these QDs are shown in Fig. 1. The observed results in Fig. 1 indicate that all CdSe1, CdSe2, and CdSe3 QDs have spherical shapes and uniform distributions with sizes ∼ of 3.5, 4.7, and 5.8 nm, respectively.

**Fig. 2:**
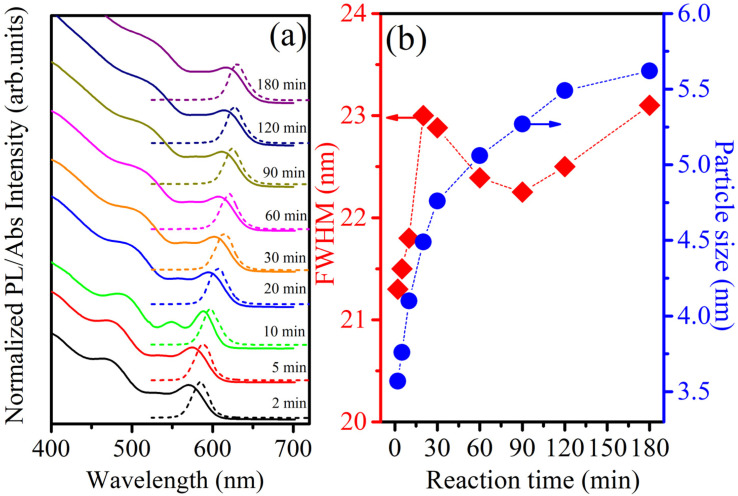
(a) UV-vis absorption and PL spectra of CdSe QDs with *λ*_ex_ = 355 nm, (b) dependence of FWHM and particle size of CdSe QDs on fabrication time.

**Fig. 3:**
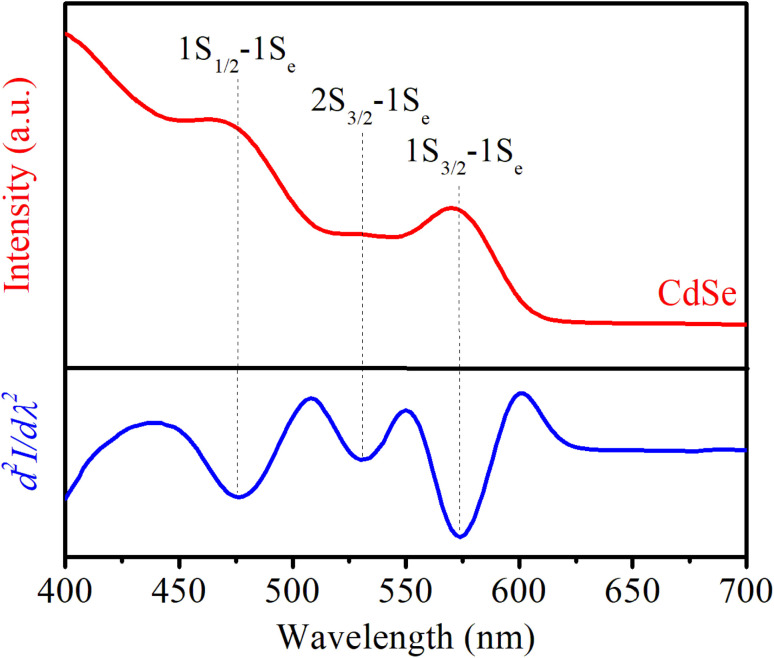
UV-visible absorption spectrum of CdSe QDs and its quadratic derivative.

Steady-state PL and UV-vis absorption spectra (corresponding to the dotted and solid lines, respectively) of the CdSe QDs (reaction time from 2 to 180 min) are shown in Fig. 2a. For the absorption spectrum, we can see clear excitonic absorption peaks, which proves that CdSe QDs have a narrow size distribution.^24^ With increasing reaction times, these absorption peaks moved to longer wavelengths due to the increasing particle size. Similar to the absorption spectra, the PL spectra of the CdSe QDs red-shifted from 585 to 629 nm as the fabrication time increased from 2 to 180 min. They are all symmetrical, narrow, and have no emission of surface states or defects. The size of CdSe QDs can be determined through the wavelength (*λ*) of the excitonic absorption peak, which can be determined from the second derivative method of the absorption spectrum.^24,25^ The second derivative method is a useful technique for analyzing absorption spectra, particularly when investigating exciton states in materials. Excitons are bound electron–hole pairs created when a material absorbs photons, and their energy states can be challenging to pinpoint using traditional absorption spectra due to broad peaks or overlapping transitions. By taking the second derivative of the absorption spectrum, this method enhances the resolution of spectral features.^26^ The three lowest energy states, 1S_3/2_–1S_e_, 2S_3/2_–1S_e_, and 1S_1/2_–1S_e,_ were accurately determined from the absorption spectra of CdSe (2 min) QDs combined with its second derivative curve, as shown in Fig. 3. The bandgap energy of CdSe QDs is determined through the first excitonic absorption peak (1S_3/2_–1S_e_) by the equation: *E*_g_ = *hc*/*λ*, where: *h* is Planck's constant (6.626 × 10^−34^ J s), *c* is the speed of light (3 × 10^8^ m s^−1^), and *λ* (m) is the wavelength of the first excitonic absorption peak. The first excitonic absorption peaks and bandgap energies of the samples are listed in Table 1.

**Table 1:** Parameters of CdSe QDs: AbS peak, PL peak, FWHM, bandgap, and size

Sample	AbS peak (nm)	PL peak (nm)	FWHM (nm)	Bandgap (eV)	Size (nm)
2 min	571.61	585.22	21.31	2.17	3.55
5 min	575.22	588.61	21.50	2.16	3.76
10 min	587.84	597.84	21.82	2.11	4.1
20 min	594.93	606.56	23.01	2.08	4.49
30 min	602.65	614.23	22.88	2.06	4.76
60 min	607.53	621.18	22.39	2.04	5.06
90 min	611.78	624.37	22.25	2.03	5.27
120 min	614.93	626.56	22.52	2.02	5.49
180 min	618.40	629.84	23.13	2.00	5.64

The Effective Mass Approximation (EMA) is used as a theoretical model to estimate the size of semiconductor CdSe QDs by accounting for quantum confinement effects. In this model, the electrons and holes within the quantum dot are treated as particles confined in a potential well, with their energy levels modified owing to the reduced size of the quantum dot compared to bulk materials. The total energy *E*_g_(*r*) of an electron–hole pair (exciton) in a QD is given by:^27^1
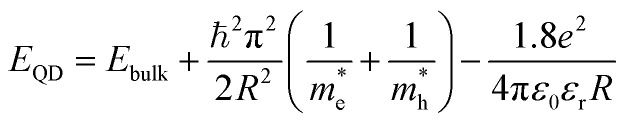
where *E*_QD_ is the first exciton absorption peak energy of the QD, *E*_bulk_ is the bandgap energy of bulk CdSe (approximately 1.74 eV at room temperature), ℏ is the reduced Planck's constant, *R* is the radius of the QDs and is given by2
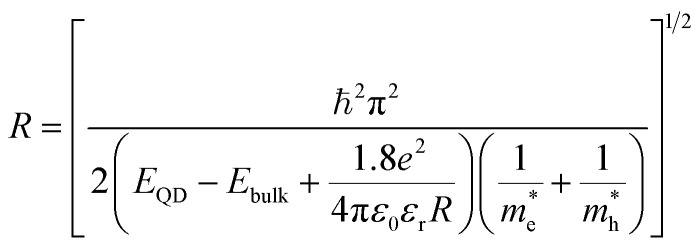

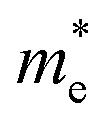
 is the effective mass of the electron (approximately 0.13*m*_0_), 
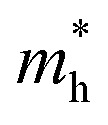
 is the effective mass of the hole (approximately 0.45*m*_0_), *e* is the elementary charge (1.6 × 10^−19^ C), *ε*_0_ is the permittivity of free space (8.854 × 10^−12^ F m^−1^), and *ε*_r_ is the relative permittivity of CdSe (approximately 9.5).^28–30^ The size (*D* = 2*R*) of CdSe QDs was determined to be in the range of 3.55–5.64 nm (Table 1).

Fig. 2b reveals that the particle size and full width at half maximum (FWHM) vary as a function of reaction time. It should be noticed that the size of CdSe QDs varies strongly in the period of 2 to 60 min and changes little for reaction times longer than 60 min. This demonstrates that the growth of QDs occurred rapidly within the first 30 min. The size of the ZnS QDs obtained from the absorption spectrum was consistent with that obtained from the TEM image (Fig. 1). The results in Fig. 2b and Table 1 show that we controlled the particle size distribution (FWHM values of 21–23 nm) during the fabrication period. Uniformly sized CdSe QDs will be advantageous for studying the effect of size on biomedical applications.

**Fig. 4:**
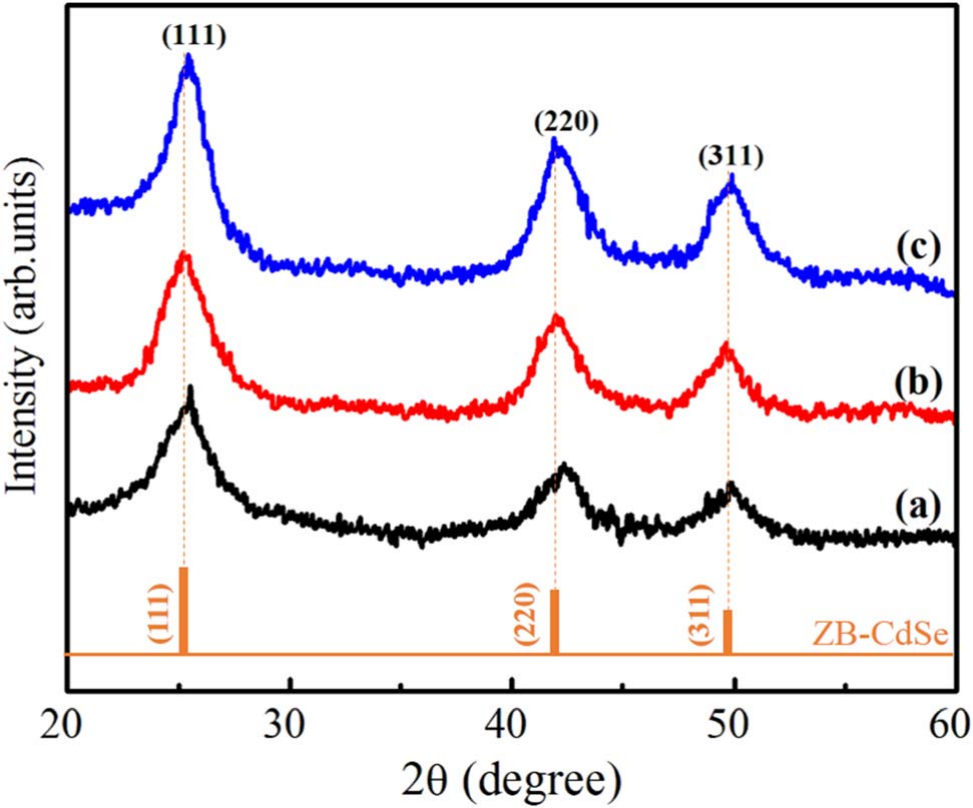
XRD of CdSe QDs at the reaction time: (a) 2 min, (b) 30 min and (c) 180 min, respectively.

Fig. 4 shows the XRD patterns of CdSe1, CdSe2 and CdSe3 QDs. The observed results in Fig. 4 show that the CdSe QDs crystallized in the cubic zinc blende (ZB) structure (space group F-43 m). The three diffraction peaks located at approximately 25.12°, 41.96° and 49.88° are assigned to (111), (220) and (311) planes of the CdSe, respectively. The CdSe or CdS QDs usually have a ZB structure when they are fabricated at low temperatures (<240 °C)^29^ and using OA ligand.^30^

**Fig. 5:**
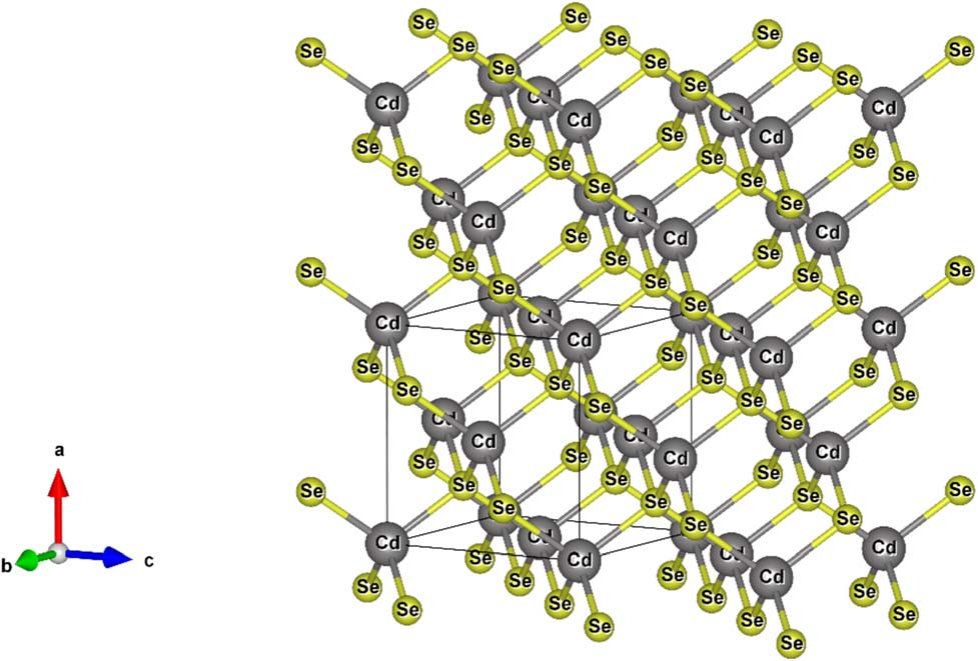
Unit-cell scheme of CdSe QDs.

X-ray diffraction (XRD) is a valuable tool for analyzing the crystal structure, phase composition, and average size of QDs. The lattice constants and crystallite sizes of the CdSe QDs were determined through the broadening of the diffraction peaks. The crystallite size of CdSe QDs can be calculated from the broadening of the diffraction peaks using the Scherrer equation:^31^3
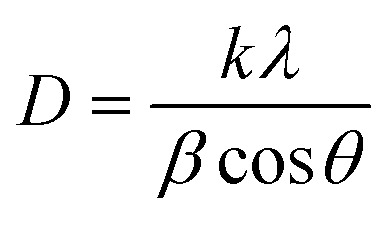
where *D* is the crystallite size, *k* is the shape factor (typically *K* = 0.9), *λ* is the X-ray wavelength (for Cu K_α_ radiation, *λ* = 1.5406 Å), *β* is the full width at half maximum (FWHM) of the diffraction peak in radians, and *θ* is the Bragg angle (the angle at which the peak occurs). The unit-cell scheme of the CdSe QDs is shown in Fig. 5. The unit cell dimension or the lattice constant (*a*) of the QDs (for the ZB structure) was calculated using the following equation:^31,32^4
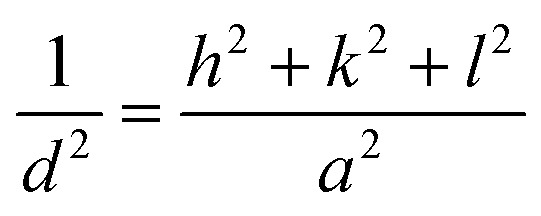
where *d* is the distance between the two planes (Å), and *h*, *k*, and *l* are the Miller indices. *d*_*hkl*_ is the interplanar spacing, which was calculated using Bragg's equation:^33,34^

5 *nλ* = 2*d*_*hkl*_ sin *θ*

where *n* is an integer (the order of the diffraction peak). In this study, the lattice parameters were determined through the (111) diffraction peak, which is the most intense peak. The stress in the host lattice (micro-strain (*ε*)) can be determined using the following formula:^33,34^

6 *ε* = (*β* cos *θ*)/4

The lattice parameters of CdSe1, CdSe2, and CdSe3 QDs were calculated and are listed in Table 2.

**Table 2:** Lattice parameters, micro-strain, and crystallite size of the obtained CdSe QDs

Sample	2*θ* (deg.)	*β* (deg.)	*d*_*hkl*_ (Å)	*a* (Å)	*D* (nm)	*ε* × 10^−3^
CdSe1	25.12	2.95	4.18	6.03	2.76	12.56
CdSe2	25.10	2.23	4.19	6.05	3.65	9.49
CdSe3	25.05	1.84	4.22	6.09	4.42	7.83

**Fig. 6:**
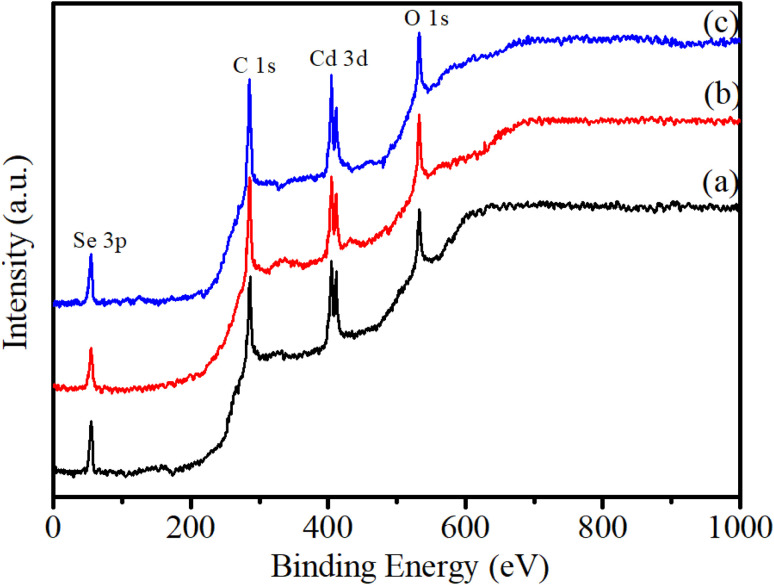
Survey XPS spectra of samples: (a) CdSe1, (b) CdSe2, (c) CdSe3.

Fig. 6 shows the survey XPS spectra of CdSe QDs. The observation results in Fig. 6 show the presence of the elements Cd, Se, C, and O at their characteristic energy positions. The spectra reveal two distinct peaks at binding energies of 404.48 eV and 411.89 eV, corresponding to the Cd 3d_5/2_ and Cd 3d_3/2_ states, respectively. Additionally, a single peak observed at 54.76 eV is attributed to the Se 3d state. The binding energies at 531.62 eV and 284.79 eV are assigned to O 1s and C 1s states, respectively, which suggest the presence of these elements due to residual precursor materials on the QDs.

### 3.1 CdSe QDs reduce the viability of AGS gastric cancer cells

**Fig. 7:**
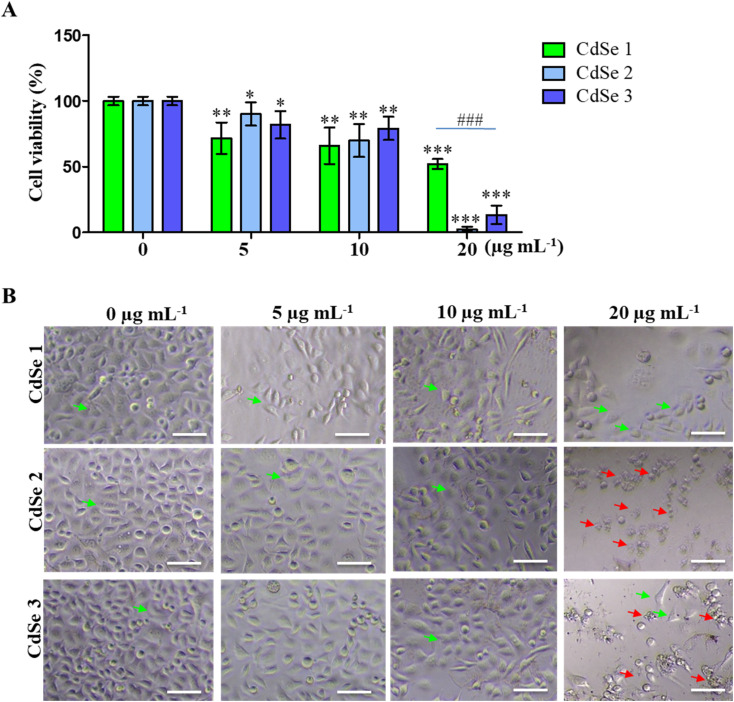
Effect of CdSe QDs on the proliferation and morphology of AGS gastric cancer cells. (A) Effect of CdSe QDs at concentrations of 5–20 μg mL^−1^ on % cell proliferation. The control (0 μg mL^−1^) was treated with an equivalent of toluene. Cell viability was determined by MTT assays (*n* = 5), *p < 0.05; **p < 0.01; ***p < 0.001 as compared to the control by Mann–Whitney test; ^###^*p* < 0.001 by one way Dunnett test. (B) Representative images of cellular morphology in AGS cells after 24 h of treatment with various concentrations of CdSe QDs. Green arrows show the morphology of live cells, and red arrows show the morphology of dead cells. Scale bars: 50 μm.

The cytotoxic effect of CdSe QDs on AGS gastric cancer cells was assessed using the MTT assay. As presented in Fig. 7A, CdSe QDs significantly inhibited the growth of AGS cells in a dose-dependent manner. At concentrations ranging from 5 to 20 μg mL^−1^, CdSe1 reduced cell viability from 71.67 ± 13.45% to 52.18 ± 4.18% compared to the control (100 ± 3.43%). After 24 h of treatment with CdSe2, cell viability ranged from 90.16 ± 9.95% to 2.35 ± 2.13%. For CdSe3, the live cell rate was measured as 81.83 ± 11.74% to 13.49 ± 7.82%, respectively. The impact of CdSe QDs on cancer cell morphology is also illustrated in Fig. 7B, where changes in cell morphology were observed at a concentration of 20 μg per mL CdSe QDs. Notably, almost all cells died when treated with 20 μg per mL CdSe2. Thus, the CdSe QDs synthesized in this study demonstrated cytotoxic ability against AGS gastric cancer cells, with CdSe2 exhibiting stronger toxicity than CdSe1 and CdSe3.

Previous reports have also highlighted the ability of carbon quantum dots to induce cell death in breast cancer cells,^35,36^ and liver cancer cells.^37^ Notably, a recent study indicated that ZnO QDs could inhibit the proliferation of breast cancer stem cells,^38^ and act as carriers for targeted drug delivery to cancer cells.^39^ The ability of CdSe QDs to inhibit HepG2 liver cancer cells at concentrations ranging from 7 to 14 μg mL^−1^ has been documented.^40^ The formation of functional groups on the surface of CdSe QDs during synthesis has been demonstrated in previous studies, particularly the COO–, O–H, and thiol groups.^41,42^ These functional groups exhibit mild acidity and can participate in redox reactions, and they have been shown to inhibit the formation and proliferation of cancer cells.^43^ Moreover, these functional groups facilitate easier cellular uptake and counteract membrane efflux pumps, making them applicable in the development of metal nanocomplexes and drug-delivery quantum dots.^44^ The inhibitory effect of CdSe QDs on gastric cancer cells synthesized in this study suggests that QDs not only find applications in cancer imaging but also have the potential for cytotoxicity and destruction of cancer cells.

### 3.2 CdSe QDs arrest the cell cycle of AGS gastric cancer cells

**Fig. 8:**
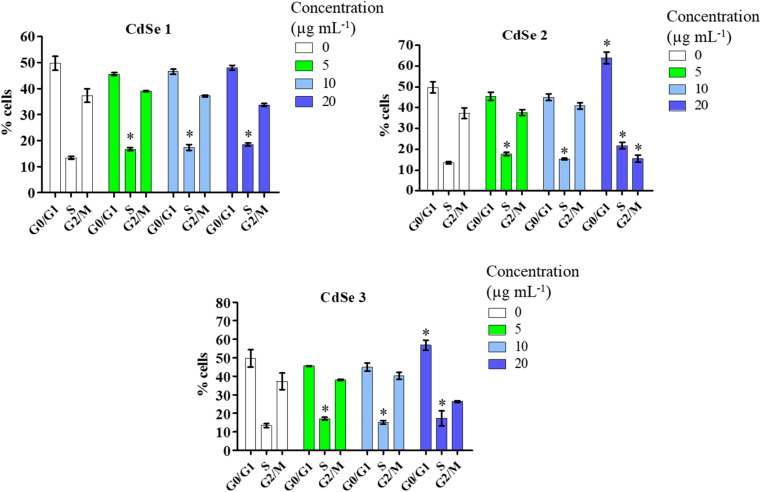
Effect of CdSe QDs on the cell cycle of AGS gastric cancer cells. AGS cells were stained with propidium iodide and analyzed by flow cytometry after 24 h of treatment with CdSe QDs at 5–20 μg mL^−1^. Data are represented as the mean and interval of values (*n* = 3). Mann–Whitney test, *p < 0.05 *versus* control.

To test the hypothesis that CdSe QDs interfered with the cell cycle, leading to the arrest of AGS cell division, a cell cycle analysis using flow cytometry was conducted. The results of the analysis (Fig. 8) showed that all three synthesized forms of CdSe QDs induced significant changes in the cell cycle phase S compared to the control (*p* < 0.05). Notably, CdSe2 and CdSe3 QDs caused cell cycle arrest in the G0/G1 phase when AGS cells were treated with these CdSe QDs at a concentration of 20 μg mL^−1^, with percentages of 63.93 ± 4.91% and 56.83 ± 2.75%, respectively, compared to 49.70 ± 4.68% in the control. The loss of control over the cell division cycle is considered an important mechanism leading to the development of cancer cells. One of the significant approaches to developing anticancer drugs today is to target the proteins that regulate the cell cycle, causing cells to undergo disruptions in the division process, resulting in the cessation of uncontrolled proliferation of cancer cells.^45^ Previously, the impact of carbon quantum dots,^46^ graphene quantum dots,^47^ or cadmium telluride quantum dots^48^ on cell cycle arrest in the G2/M phase of MCF7 breast cancer cells and HepG2 liver cells^49^ has also been reported. The influence of QDs on the expression of cell cycle regulatory proteins such as P21, P27,^47^ or P53^49^ has been documented. QDs enhance the expression of P21 and P27 proteins, leading to cell cycle arrest in the G2/M phase in MCF-7, MDA-MB-231, and T-47D breast cancer cells.^47^ Here, we demonstrated that CdSe QDs increased the percentage of gastric cancer cells in the S or/and G0/G1 phases, while reducing the percentage of cells in the G2/M phase. Thus, it can be observed that QDs may induce cell cycle arrest at different phases, depending on their properties.

### 3.3 CdSe QDs induce apoptosis in AGS gastric cancer cells

**Fig. 9:**
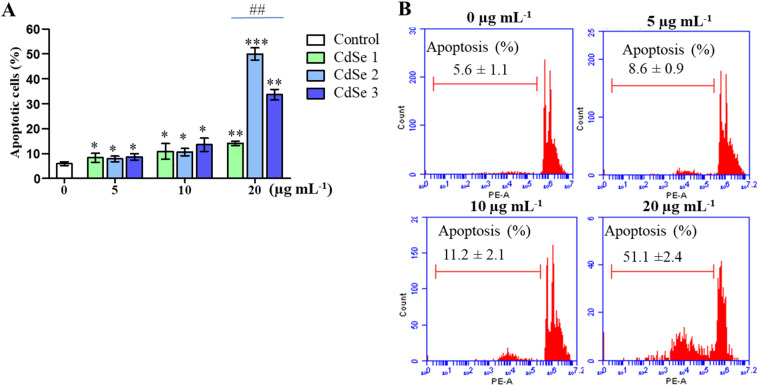
Effect of CdSe QDs on the apoptosis of AGS gastric cancer cells. AGS cells were treated with CdSe QDs at concentrations ranging from 5 to 20 μg mL^−1^. The control group (0 μg mL^−1^) was treated with an equivalent amount of toluene, and apoptosis analysis was performed using flow cytometry. (A) The histogram presents the distribution of apoptosis in AGS cells treated with various concentrations of CdSe QDs compared to the control. *p < 0.05, **p < 0.01, ***p < 0.01 *versus* control; Mann–Whitney test. ^##^*p* < 0.01, one-way Dunnett's test. (B) The apoptosis rate was measured using flow cytometry when cells were treated with CdSe2 (*n* = 3).

The impact on the cell cycle not only impairs cell proliferation, but also promotes apoptosis. To assess the effect of CdSe QDs on apoptosis, cells treated with different concentrations of QDs were analyzed using flow cytometry. As illustrated in Fig. 9, CdSe QDs increased the number of apoptotic cells in a dose-dependent manner. More importantly, at a concentration of 20 μg mL^−1^, CdSe2 significantly elevated the proportion of apoptotic cells to 51.1 ± 2.4%, compared to 14.08 ± 0.90% in CdSe1 and 33.6 ± 2.11% in CdSe3 (*p* < 0.01). This result indicates that CdSe2 not only arrests the cell cycle at the G0/G1 phase but also induces apoptosis in AGS cells. The apoptotic effects of QDs have been assessed as a potential approach for cancer treatment.^50^ Quantum dots can activate the expression of a variety of genes related to apoptosis, such as caspase 3, caspase 7,^51^ caspase 8, caspase 9,^52,53^ and Bcl2.^52^ Notably, the upregulation of certain genes simultaneously involved in cell cycle control and apoptosis sensitivity has also been observed when breast cancer cells were treated with QDs.^47^ The mechanism of apoptosis induced by metal nanoparticles and quantum dots, which has been widely discussed recently, is closely related to the promotion of ROS generation, which leads to the destruction of cellular DNA and induction of cell apoptosis.^54^

### 3.4 CdSe QDs increase the ROS generation

**Fig. 10:**
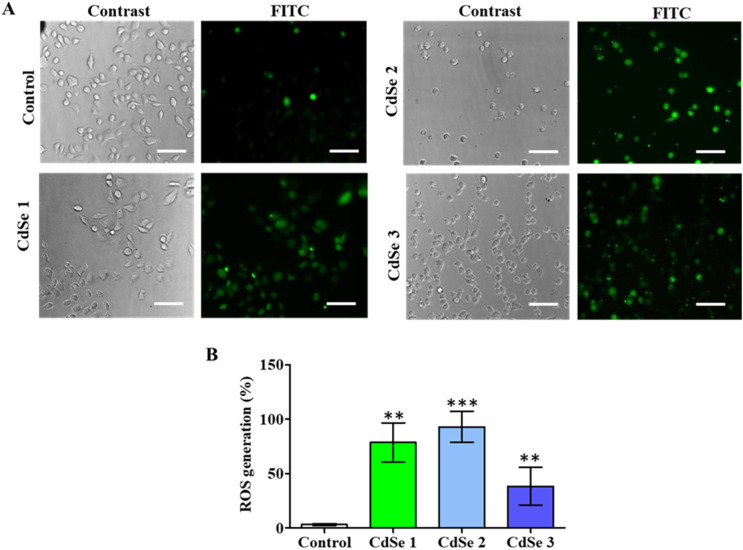
Effect of CdSe QDs on ROS production. AGS cells were treated with CdSe1, CdSe2, and CdSe3 at a concentration of 20 μg mL^−1^ for 24 h. Subsequently, they were incubated for 30 min in a DC-FDA solution (10 μg mL^−1^). (A) Cell images were recorded using the contrast phase for the identification of total cells and the FITC filter for the identification of ROS generation (green). The scale bar was set at 20 μm. Scale bar: 20 μm. (B) The change in % ROS generation of AGS cells treated with 2.0 μg mL^−1^ of CdSe QDs. Mann–Whitney Test, *p < 0.05; **p < 0.01; ***p < 0.001 as compared to control (0 μg mL^−1^).

To further elucidate the potential mechanism associated with the cytotoxic effects of CdSe QDs, cell staining using H2-DCFDA was performed to evaluate the generation of ROS induced by QDs in cancer cells (Fig. 10). Fluorescence microscopy analysis revealed that all three CdSe QDs induced the generation of reactive oxygen species (ROS) in cancer cells. The green fluorescent cells are the result of the transformation of H2DCFDA into DCFA under the influence of free radicals such as H_2_O_2_, OH–, and O_2_–. The green fluorescence cell rate significantly increased compared to that of the control for all three synthesized CdSe QD forms (Fig. 10A). Notably, CdSe2 induced 92.96 ± 14.06% of ROS-expressing cells.

The positive cell rates with ROS when treated with CdSe1 and CdSe3 were 78.65 ± 17.98% and 38.37 ± 17.33%, respectively, compared to 3.11 ± 0.93% in the control (*p* < 0.01). This is a common characteristic of metal nanoparticles when acting on cancer cells. The generation of ROS has been linked to cell membrane destruction,^55^ DNA breakage in cells,^56^ induction of cell cycle arrest,^57^ initiation of apoptosis,^58^ and ultimately the inhibition of cell proliferation.^59^ In addition, recent evidence has indicated the enhanced presence of ROS in cancer cells, promoting cellular senescence and arresting irreversible cell division, leading to the inhibition of tumor growth.^60,61^ Therefore, inducing the generation of a significant amount of ROS in cells is a promising approach in the development of modern cancer therapies. Our findings contribute to elucidating the anticancer potential of CdSe QDs.

One of the current challenges in the application of CdSe QDs in living organisms is their toxicity and biocompatibility. CdSe QDs have been reported to exhibit toxicity to various organs, including the liver, kidneys, and lungs.^62^ Exploiting their cytotoxic properties against cancer cells while mitigating their adverse effects on healthy tissues is a topic of significant interest. To address these limitations, it is essential to explore different delivery systems, such as polymer encapsulation, lysosomal targeting, or silica coating for CdSe QDs,^63^ as well as to adjust dosage and particle size.^62^ Furthermore, the conjugation of specific monoclonal antibodies to the surfaces of these particles may be considered to facilitate direct targeting of the intended tissues without adversely affecting other cells in the body.^64^

In addition to their reported optical properties used in medical imaging, CdSe QDs can be developed into nanomaterials for therapeutic applications, particularly in cancer treatment. Ongoing research will focus on structural modifications to minimize side effects and enhance targeted efficacy against cancer cells, while clinical trials and evaluations will also receive considerable attention.^65^

## 4 Conclusions

CdSe QDs with the size in the range 3.5–5.8 nm and a ZB crystal structure were successfully synthesized using the wet chemical method. The fabricated CdSe QDs have a narrow size distribution (below 23 nm), strong emission and emission peak ranging from 585 to 630 nm. The anti-cancer properties of CdSe QDs were examined in HepG2 liver cancer cells using various methods, including cell viability screening (MTT assay), as well as cell cycle and apoptosis analysis *via* flow cytometry. Reactive oxygen species (ROS) production was measured using the cell fluorescence staining technique with H2DCFDA. Three different sizes of CdSe QDs: CdSe1 (3.5 nm), CdSe2 (4.7 nm), and CdSe3 (5.8 nm) were chosen to assess their impact on the destruction of stomach cancer cells. All CdSe QDs demonstrated potential toxicity to cells at concentrations between 5 and 20 μg mL^−1^. The synthesized CdSe QDs have been shown to arrest the cell cycle in the S and G0/G1 phases, inducing apoptosis through the generation of ROS in cells, with CdSe2 QDs exhibiting stronger cell-inhibitory activity against AGS cells than CdSe1 and CdSe3. CdSe QDs have demonstrated potential for the development of therapeutic approaches for gastric cancer cells.

## Data availability

The data supporting this study's findings are available on request from the corresponding author [Nguyen Thi Minh Thuy, email: thuyntm@tnue.edu.vn]. The data are not publicly available due to [reason, privacy].

## Author contributions

L. T. T. Huong: conceptualization, data curation, writing – original draft. N. T. Ha: data curation. N. P. Hung: supervision, data curation. N. X. Ca: conceptualization, investigation, writing – original draft. N. T. Hien: investigation, software. N. T. Luyen: data curation. N. T. M. Thuy: conceptualization, writing – original draft, writing – review & editing.

## Conflicts of interest

There are no conflicts to declare.
